# Development and Application of a Robust Imine-Based Covalent Organic Framework for Stir Bar Sorptive Extraction of Estrogens in Environmental Water

**DOI:** 10.3390/molecules29235763

**Published:** 2024-12-06

**Authors:** Jianing Sun, Xixi Lian, Lianzhi Wang, Zhengchao Duan

**Affiliations:** School of Chemistry and Environmental Engineering, Hubei Minzu University, Enshi 445000, China; sjn_ing@163.com (J.S.); liana190203@163.com (X.L.)

**Keywords:** covalent organic framework, estrogens, stir bar sorptive extraction, high-performance liquid chromatography, environmental water samples

## Abstract

A covalent organic framework (COF) based on imine was synthesized using 2,5-dihexoxyterephthalaldehyde (DHT) and 1,3,5-tris(4-aminophenyl) benzene (TAPB) as starting materials. The TAPB-DHT-COF exhibited satisfactory chemical stability, making it a promising adsorbing material for stir bar sorptive extraction (SBSE) of four estrogens, including estrone (E1), β-estradiol (E2), hexestrol (HES), and mestranol (MeEE2), in ambient water samples. The extracted analytes were subsequently analyzed using a high-performance liquid chromatography-diode array detector (HPLC-DAD). A series of parameters affecting the SBSE process, such as solution pH, ionic strength, extraction time, and desorption solvent, were investigated by the controlled variable method. Under optimal conditions, the limit of detection (LODs) for the four targeted estrogens ranged from 0.06 to 0.15 µg/L, with a linear range from 0.2 to 100 µg/L. The observed enrichment factor (EF) ranged from 39 to 49, while the theoretical EF was estimated to be 50-fold. This methodology can be applied to the identification of estrogens in three environmental water samples.

## 1. Introduction

Estrogens are steroid hormones that play a crucial role in maintaining female secondary sexual characteristics, regulating bone density, and protecting the cardiovascular and central nervous systems [1]. They are characterized by their aromatic ring structure. Natural estrogens found in the body include estrone (E1), β-estradiol (E2), and estriol (E3), while synthetic estrogens include hexestrol (HES), stilboestrol, nilestrol, ethinyl estradiol, and mestranol (MeEE2) [2,3]. However, estrogens have been detected in water and other environments, posing a threat to organisms by interfering with the endocrine system and disrupting normal hormone metabolism. This can lead to diseases such as prostate cancer, breast cancer, and fibroids [4,5,6]. Therefore, it is of great significance to develop a straightforward, precise, and highly sensitive technique for the analysis of environmental estrogens [7].

Detection methods for estrogens currently include high-performance liquid chromatography (HPLC), gas chromatography (GC), capillary electrophoresis (CE), and enzyme-linked immunoassay (ELISA) [8,9,10,11]. HPLC is widely used due to its reproducibility and automation capabilities [12,13]. However, direct detection of low concentrations of estrogens in complex environmental samples using HPLC is challenging, thus requiring sample pretreatment prior to analysis [14,15]. Conventional pretreatment techniques, such as liquid-liquid extraction (LLE) and solid-phase extraction (SPE), often involve extensive solvent usage and time-consuming procedures [16,17]. Alternatively, new sample pretreatment techniques such as magnetic solid-phase extraction (M-SPE), solid-phase microextraction (SPME), and stir bar sorptive extraction (SBSE) have emerged, offering cost-effectiveness and ease of operation while minimizing solvent wastage [18,19,20]. Among these techniques, SBSE stands out for its low solvent consumption and the absence of additional magnetic stirring requirements [21,22].

In 1999, Baltussen et al. introduced the concept of stir bar sorptive extraction (SBSE) [23]. This technique has since been successfully applied to the analysis of various trace organic compounds in complex sample matrices. However, SBSE suffers from limited availability of extraction coatings and insufficient extraction efficiency [24]. Currently, the market offers three types of coated stir bars: polyacrylate, ethylene glycol-silicone, and polydimethylsiloxane (PDMS) [25,26,27]. While these coatings are suitable for adsorbing non-polar and weakly polar chemicals, they are less effective for polar and moderately polar substances [28]. Hence, there is a critical need to develop new stir bar coating materials that can accommodate different polarity analyses.

Covalent organic frameworks (COFs) are a class of porous materials composed of organic units (such as C, H, O, N, and other elements) that are interconnected through covalent bonds [29]. These materials possess several advantages over traditional polymers, including a porous structure, high surface area, and excellent stability, which afford them a large number of adsorption sites and a high adsorption capacity for various targets [20]. Moreover, COFs exhibit high thermal stability, low density, adjustable pore size, and tunable functionalization [30]. The presence of diverse functional groups enables COFs to interact with targets through various forces, such as hydrophobic interactions, π–π interactions, and hydrogen bonding [31]. Given these unique characteristics, researchers can select appropriate COF materials as solid-phase microextraction (SPME) coatings to extract different analytes from various sample matrices according to specific application requirements.

This is based on the fact that all the selected estrogen objects contain benzene ring structures and hydroxyl groups. At the same time, their logP is low and they have good fat solubility. Therefore, we chose COF materials with a multi-benzene ring structure as the adsorption material for SBSE. Meanwhile, appropriate long chains were introduced into its pores to utilize the π–π interaction between COF materials and estrogens, the hydrogen bonding interaction between hydroxyl and carbonyl groups and the adsorption material, and the hydrophobic effect of the long-chain structure in the COF pores on estrogen samples to improve the adsorption and extraction efficiency of estrogen samples. Thus, the detection limit for the analysis of estrogen objects in the SBSE process was improved, and it can be applied to the detection of environmental samples with low content.

The objective of this research was to synthesize a COF material using 2,5-dihexoxyterephthalaldehyde (DHT), which contains n-hexyloxy side chains and 1,3,5-tris(4-aminophenyl) benzene (TAPB) as monomers. We anticipated that the adsorption of the target estrogens onto the synthesized COFs would primarily occur through π–π interactions between the benzene rings of the COF and estrogens. Additionally, hydrogen bonding was expected to take place between the oxygen atoms of the COF’s side chains and the hydroxyl groups of the estrogens. Moreover, we anticipated that the hydrophobic interaction between the long-chain alkyl group of the COF and the estrogens would also contribute to the adsorption process.

## 2. Results and Discussion

### 2.1. Preparation and Characterization of TAPB-DHT-COF

#### 2.1.1. Influence of Catalyst Amount on the Morphology of TAPB-DHT-COF Materials During the Preparation Process

**Figure 1:**
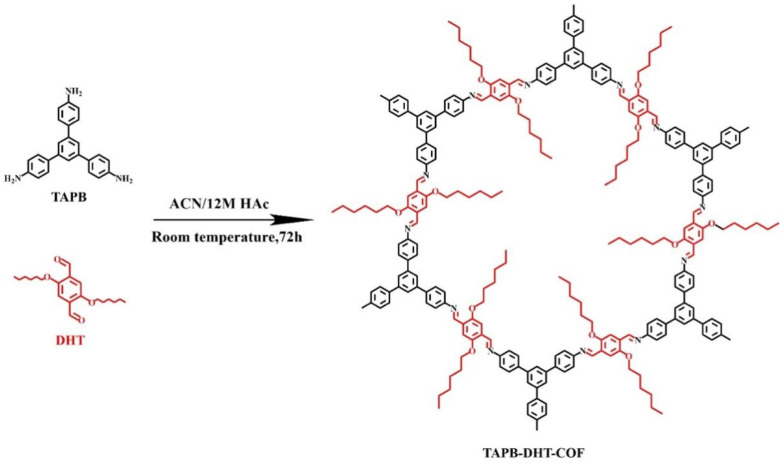
Synthetic route of TAPB-DHT-COF.

**Figure 2:**
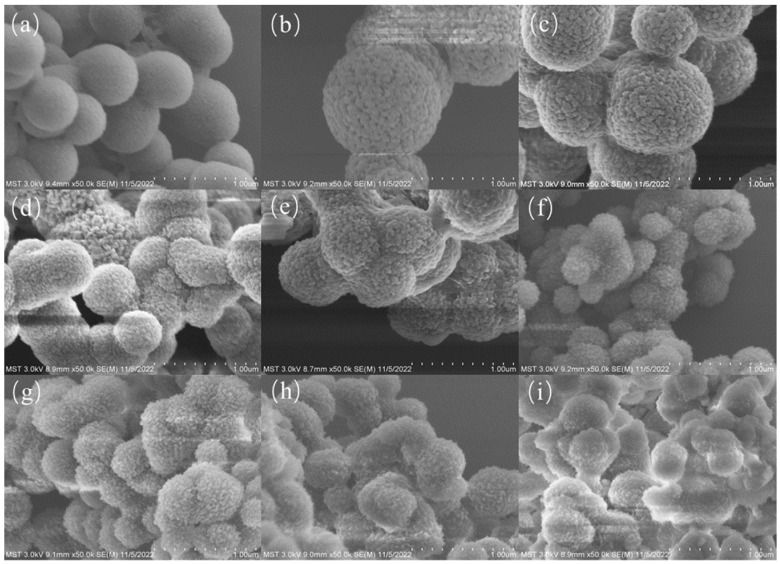
Impact of catalyst amount (HAc: (**a**) 0.2 mL, (**b**) 0.4 mL, (**c**) 0.6 mL, (**d**) 0.8 mL, (**e**) 1.0 mL, (**f**) 2.0 mL, (**g**) 3.0 mL, (**h**) 4.0 mL, and (**i**) 5.0 mL) on TAPB-DHT-COF structure revealed by SEM images.

The TAPB-DHT-COF synthetic technique was taken from the literature [32] and a little modified (Figure 1). In brief, a 50 mL centrifuge tube was preloaded with TAPB (28.1 mg, 0.08 mmol) and DMTP (40.1 mg, 0.12 mmol). Then, 10 mL ACN was spiked into the mixture, followed by ultrasonic mixing for 60 s. After that, 0.4 mL acetic acid (12 mol/L) was added, and the centrifuge tube was vortexed for 30 s. Finally, the mixture was maintained at room temperature for three days. The obtained precipitate was rinsed by THF (3 × 20 mL) and anhydrous ethanol (3 × 20 mL) successively, and dried under vacuum at 60 °C overnight, yielding yellow powder (56 mg, 82%). The amount of catalyst plays a significant role in influencing the reaction of Schiff bases. In this study, the synthesis reaction of TAPB-DHT-COF was catalyzed by 12 M acetic acid, and the impact of different catalyst amounts on the product structure was investigated. SEM images of the TAPB-DHT-COF obtained with various catalyst additions are shown in Figure 2.

Observations revealed that when 0.2 mL of catalyst was used, the morphology of the TAPB-DHT-COF spheres appeared smooth, which is not favorable for adsorption. However, when 0.4 mL of catalyst was added, the spherical shape of the TAPB-DHT-COF became regular and showed an obvious porous structure, providing a sufficient number of adsorption sites. On the other hand, the addition of 0.6 mL of catalyst resulted in different particle poles in the TAPB-DHT-COF. Further increase in the amount of catalyst led to irregular changes in the morphology of the TAPB-DHT-COF, along with adhesion phenomena.

Based on these experimental results, it is clear that a lower dosage of catalyst favors the formation of a homogeneous pole and individual independent spherical morphology in the TAPB-DHT-COF. However, the smooth surface of the formed TAPB-DHT-COF hinders its adsorption ability. Conversely, higher amounts of catalyst result in irregular morphology and adhesion of the TAPB-DHT-COF, which also negatively affects adsorption. Therefore, the amount of catalyst significantly impacts the morphology of the TAPB-DHT-COF, and an optimal amount is required for its proper formation.

In subsequent experiments, 0.4 mL of 12 M acetic acid was chosen as the catalyst for the synthesis of TAPB-DHT-COF.

#### 2.1.2. Characterization of TAPB-DHT-COF: FT-IR, XRD, TG, Nitrogen Adsorption–Desorption, and SEM Analysis

**Figure 3:**
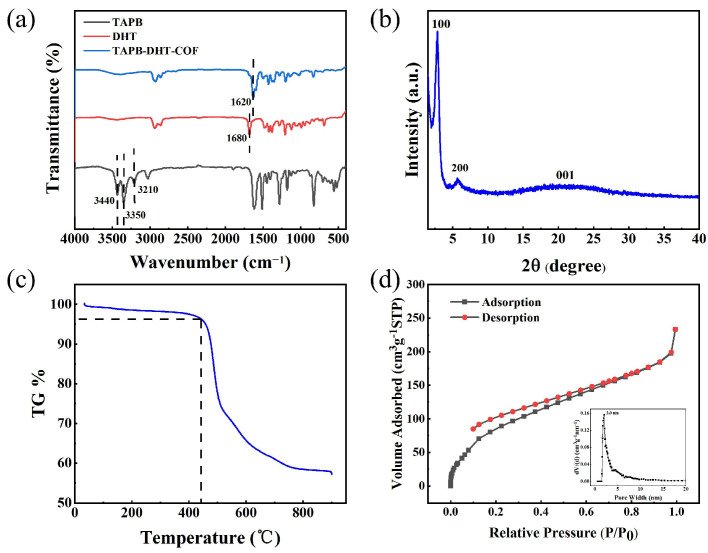
Characterization of TAPB-DHT-COF through FT-IR (**a**), XRD (**b**), TG (**c**), and nitrogen adsorption–desorption (**d**) analyses.

Fourier-transform infrared (FT-IR) spectroscopy was employed to examine the monomer and TAPB-DHT-COF structures. The obtained results are presented in Figure 3a. The FT-IR spectrum of TAPB showed prominent peaks at wavenumbers of 3440 cm^−1^, 3350 cm^−1^, and 3210 cm^−1^, which definitively corresponded to the stretching vibrations of the N-H functional group. In the case of DHT, a peak at around 1680 cm^−1^ was observed, suggesting the presence of stretching vibrations associated with the C=O functional group. Interestingly, no discernible stretching vibrations of N-H or C=O groups were observed in the FT-IR spectrum of the TAPB-DHT-COF. However, a distinctive absorption peak attributed to the C=N stretching vibration was observed at 1620 cm^−1^, further confirming the successful synthesis of this specific COF.

The crystal structure of the TAPB-DHT-COF was examined through X-ray diffraction (XRD) spectroscopy, and the findings are presented in Figure 3b. The prominent absorption peak observed at a diffraction angle of 2.84° corresponds to the (100) plane, while the absorption peak at a diffraction angle of 5.72° corresponds to the (200) plane. These peaks exhibit sharp and well-defined shapes, indicating that the TAPB-DHT-COF possesses a highly crystalline structure similar to what has been reported in the literature [33]. This observation suggests that the synthesized COF has a favorable crystal arrangement.

Thermogravimetric analysis (TG) was conducted to evaluate the thermal stability of the TAPB-DHT-COF material, as depicted in Figure 3c. The thermogravimetric curve indicates that TAPB-DHT-COF maintained its relative structural integrity up to 443 °C. As the temperature continued to rise, the COF structure ultimately collapsed and decomposed [34]. These TG results highlight the commendable thermal stability of the material.

The surface area and porous characteristics of TAPB-DHT-COF were evaluated using nitrogen adsorption–desorption isotherms. The isotherm displayed a characteristic type II adsorption pattern (Figure 3d) in accordance with the IUPAC standard, indicating a mesoporous nature with an H3-type hysteresis loop [35]. The surface area of TAPB-DHT-COF was determined to be 381 m^2^·g^−1^, while the total pore size was measured at 2.0 nm. These findings highlight the significant surface area and uniform porosity exhibited by TAPB-DHT-COF.

#### 2.1.3. Morphological Characterization of COF-Coated Stir Bar

**Figure 4:**
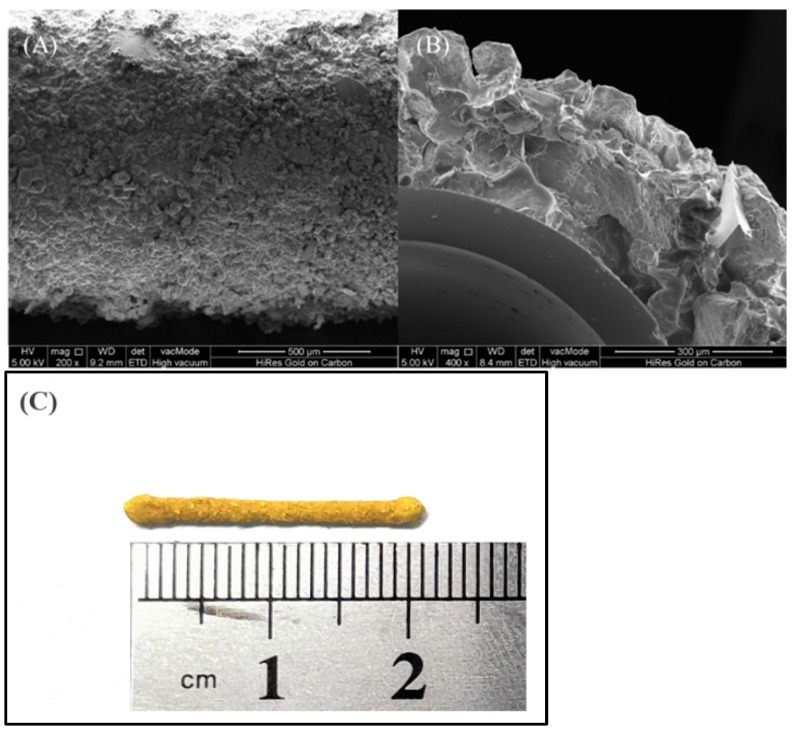
Morphology and thickness analysis of TAPB-DHT-COF-coated stir bar: (**A**) SEM images of the surface at 200×; (**B**) cross-section at 400×; (**C**) full view of stir bar.

The morphology and thickness of the stir bar coated with TAPB-DHT-COF were examined using scanning electron microscopy (SEM), as presented in Figure 4. The SEM images revealed a surface of the stir bar coated with TAPB-DHT-COF characterized by loose porosity. Furthermore, the estimated thickness of the TAPB-DHT-COF coating was approximately 289.7 µm.

### 2.2. Optimization Strategies for Enhanced Extraction and Analysis of Endocrine Disrupting Compounds

The COF-coated stir bars were prepared according to a previously published method [36]. The stir bar sorptive extraction conditions, such as desorption solvent, desorption volume, stirring rate, extraction time, desorption time, salt concentration and pH, were optimized.

#### 2.2.1. Optimal Desorption Solvent

In order to achieve optimal desorption efficiency and ensure complete desorption of the target analyte from the stir bar coating, it is crucial for the desorption solvent to have a greater affinity for the target analyte than the adsorbent material. Given that the target estrogens all contain hydroxyl groups in their chemical structures, it is more desirable to use polar solvents for their desorption based on the principle of similar solubility.

The four target estrogens possess low polarity (log P of 3.68–5.17) and poor water solubility, and therefore organic solvents were considered for desorption. Because estrogens have good solubility in methanol, methanol was chosen as the desorption solvent. We also examined the effect of adding an appropriate amount of water or NaOH aqueous solution to methanol on the analysis results. The results are shown in Figure S1. From the results in the figure, it can be seen that methanol had a good analytical effect, but its analytical efficiency decreases when water or NaOH solution was added. Therefore, methanol will be used as the resolving agent in subsequent studies.

#### 2.2.2. Optimizing Desorption Volume

The desorption volume was optimized using methanol as the solvent. Figure S2 shows the comparison of peak regions corresponding to the target estrogens during the first and subsequent desorption processes using different desorption volumes (200 µL, 250 µL, 300 µL, 350 µL, 400 µL, and 450 µL). It was observed that even with a low volume of methanol, the efficiency of the second desorption of the target analytes remained high. However, when the desorption volume was increased to 450 μL, all the target analytes’ desorption efficiencies in the second desorption solvent were found to be less than 10% of those in the first desorption solvent. To ensure a larger enrichment factor, thus, for subsequent experiments, 450 µL of methanol was chosen to desorb the four target estrogens in order to ensure a high-efficiency factor and reduce the limit of detection of the method.

#### 2.2.3. Optimizing Stirring Rate

Stirring can accelerate the mass transfer rate of the target analytes and reduce the extraction equilibrium time. In this study, the effect of the stirring rate on the adsorption efficiency of four estrogens was investigated, with a range of stirring rates from 400 to 900 rpm. Figure S3 shows that the extraction efficiency increased progressively as the stirring rate was increased within the range of 400–700 rpm. A stirring rate of 700 rpm was selected for subsequent experiments to ensure optimal adsorption efficiency while avoiding potential damage to the coating on the stir bar due to vigorous stirring.

#### 2.2.4. Optimizing Extraction Time

The SBSE adsorption method is an extraction technique based on equilibrium principles. To expedite the attainment of quick equilibrium and enhance the adsorption of the desired estrogens, a stirring approach was employed in this study. The extraction duration was found to significantly impact the effectiveness of the extraction process (Figure S4). The extraction efficiency of estrogens showed an increasing trend as the extraction time ranged from 10 to 30 min, reaching equilibrium at the 30 min mark. However, extending the extraction time to 40 min did not yield any significant improvements in the extraction efficiency. Throughout this extended period, the peak areas of the estrogens remained relatively stable. Based on these findings, a 30 min extraction time was determined to be the optimal duration for the final extraction process.

#### 2.2.5. Optimal Desorption Time

In the desorption process, methanol was utilized as the adsorbent, and the desorption time was optimized within the range of 5–25 min to achieve efficient and complete desorption in a shorter duration (Figure S5). The desorption efficiency exhibited a progressive increase from 5 to 15 min. However, after the 15 min mark, the peak areas of the four target estrogens remained relatively constant. This suggests that sufficient contact between the desorption solvent and the target analytes on the adsorbent material was achieved within this time frame. In contrast, when the desorption time was too short, inadequate contact hindered effective desorption. Therefore, a desorption time of 15 min was deemed appropriate for subsequent experiments.

#### 2.2.6. Effect of Salt Concentration on Extraction Efficiency of Target Estrogens

In this study, the impact of salt concentration on the extraction efficiency of the four target estrogens was investigated. Sodium chloride (NaCl) was added to the sample solution in varying amounts (ranging from 0% to 25% (*m*/*v*)) to adjust the ionic strength of the solution. Figure S6 demonstrates that the peak areas of E1 and E2 remained relatively stable regardless of the salt concentration. This suggests that changes in the NaCl concentration did not significantly affect the solubility of these estrogens. However, it was observed that the extraction recovery of HES and MeEE2 decreased as the salt concentration increased. This phenomenon can be explained by the increase in solution viscosity, which, in turn, reduces the mass transfer rate of estrogen. Consequently, the adsorption of estrogen onto the stir bar coating was hindered. Hence, there was no need to modify the salt content in subsequent studies

#### 2.2.7. Effect of pH on Extraction Efficiency of Target Estrogens

Due to the ionizability of the target estrogens in aqueous solutions, the investigation focused on evaluating the extraction effectiveness of the TAPB-DHT-COF stir bar for these compounds. The study involved varying the pH levels of the samples from 3.0 to 12.0. The results presented in Figure S7 demonstrate that pH levels in the range of 3.0–10.0 had no significant impact on the extraction efficiency of the estrogens. However, a notable decrease in extraction efficiency for the four target estrogens was observed when the pH exceeded 10. This decrease can be attributed to the ionization of the target estrogens, which leads to increased solubility in aqueous solutions. Therefore, the extraction efficiency decreases accordingly. The pKa values of the target estrogens (Table S1) also support this observation. Since the TAPB-DHT-COF-coated stir bar was able to effectively extract the target estrogens without requiring pH adjustment, the subsequent extractions were performed without pH modification.

### 2.3. Reproducibility of TAPB-DHT-COF-Coated Stir Bar for Target Estrogens Extraction

The reproducibility of the self-made TAPB-DHT-COF-coated stir bar for extractions of four specific target estrogens was investigated. Six stir bars from the same batch of TAPB-DHT-COF and five stir bars from different batches were chosen for this study. The extraction results are presented in Table 1. The intra-batch relative standard deviations (RSDs), calculated from the six replicates, ranged from 3.71% to 7.43%. The inter-batch RSDs, determined using the five stir bars from separate batches, varied from 4.25% to 10.8%. These findings demonstrate the high level of repeatability achieved with the preparation process of the coated stir bar.

### 2.4. Evaluation of TAPB-DHT-COF-Coated SBSE-HPLC-DAD Method for Enhanced Analysis of Specific Estrogens and Investigation of Stir Bar Lifespan

The performance of the TAPB-DHT-COF-coated stir bar sorptive extraction (SBSE) coupled with high-performance liquid chromatography-diode array detection (HPLC-DAD) method for the analysis of four specific estrogens was evaluated. Optimal adsorption and desorption conditions were determined, and the desorption solution was subjected to nitrogen purging and re-dissolution in 200 µL of methanol. This procedure aimed to improve the method’s efficiency factor (EF) and decrease the limit of detection (LOD). The results of this evaluation are presented in Table 2. The linear ranges for the four estrogens were found to be 0.2–100 µg/L with linear determination coefficients ranging from 0.9991 to 0.9997. The LOD (S/N = 3) for the four estrogens ranged from 0.06 to 0.15 μg/L. The method exhibited good reproducibility with RSDs ranging from 1.80% to 4.25% (*n* = 8, *c* = 50 µg/L). Additionally, the method showed an enrichment factor (EF) of 39–49-fold (50-fold theoretical EF) for the four estrogens.

Furthermore, the lifespan of the TAPB-DHT-COF-coated stir bars was investigated, and the results are depicted in Figure S8. The data revealed that the extraction performance of SBSE using TAPB-DHT-COF did not significantly decline after 60 iterations, indicating that the coated stir bar has the potential for at least 60 applications.

### 2.5. Enhanced Adsorption of Estrogen Using TAPB-DHT-COF-Coated Stir Bar Compared to PDMS Adhesive

The results from Figure S9 show that TAPB-DHT-COF material had a significantly higher adsorption effect on estrogen compared to PDMS adhesive. This suggests that the TABP-DHT-COF stir bar has a stronger affinity for the target estrogens compared to the PDMS. The TABP-DHT-COF stir bar’s surface has roughness, providing a large contact surface area between the target analyte and the TABP-DHT-COF coating. Additionally, there may be π-π stacking and hydrogen bonding interactions between the TABP-DHT-COF coating and estrogens, along with hydrophobic interactions. These additional interactions contribute to the increased extraction efficiency for the target analyte estrogens when using the TABP-DHT-COF-coated stir bar.

### 2.6. Speculation on Adsorption Mechanism

To elucidate the adsorption mechanism between COF materials and estrogen, molecular simulation methods were employed to speculate on the adsorption mechanism. The adsorption stability model of estrogen on COF is illustrated in Figure S10a. Estrogen molecules preferentially adsorb within the COF pores, with the aromatic rings in their structure aligned as parallel as possible to the π-wall of the COF pores, indicating a π-π stacking interaction between estrogen molecules and COF materials. Meanwhile, the hydroxyl or carbonyl groups in the estrogen structure are also located as close as possible to the π-wall of the COF. Through hydrogen bonding calculations, oxygen atoms on hydroxyl or carbonyl groups in estrogen molecules can form hydrogen bonds with COFs at distances ranging from 2.6 to 3.2. Meanwhile, the oxygen atom in the COF structure and the nitrogen atom on the imine bond can also form hydrogen bonds with the hydrogen atom on the hydroxyl group in estrogen. The adsorption energies between the four types of estrogen molecules and COF are all negative (the binding energies between COF and E1, E2, HES, and MeEE2 were calculated to be −28.50, −29.71, −30.29, and −31.58 kcal/mol, respectively), indicating that estrogen molecules are more easily adsorbed on COF materials. In molecular dynamics simulations, by fixing the rings in the COF structure but releasing the long-chain alkyl structures within the pores, it was found that during the adsorption process, the long-chain alkyl groups within the pores can change their conformation and approach estrogen molecules, forming a similar encapsulation effect (Figure S10b).

So, it can be inferred that COF materials adsorb estrogen molecules through π–π interactions, hydrogen bonding interactions, and hydrophobic interactions of long-chain alkyl groups.

### 2.7. Comparative Analysis of the Analytical Performance of the Proposed Method for Estrogen Analysis

In this study, a comparative analysis of the analytical performance of the proposed method was conducted with several estrogen analysis methods reported in the recent literature. These methods include SBSE, SPE, SPME, DLLME, IOT-SPME, and VA-ME coupled to HPLC. The results of this comparison are summarized in Table 3. Compared to the other methods, the methodology employed in this study offers several advantages, such as shorter extraction time, lower limit of detection, and lower relative standard deviations.

### 2.8. Application of TAPB-DHT-COF-Coated SBSE-HPLC-DAD Method for Analysis of Estrogens in Water Samples and Evaluation of Recovery Rates

**Figure 5:**
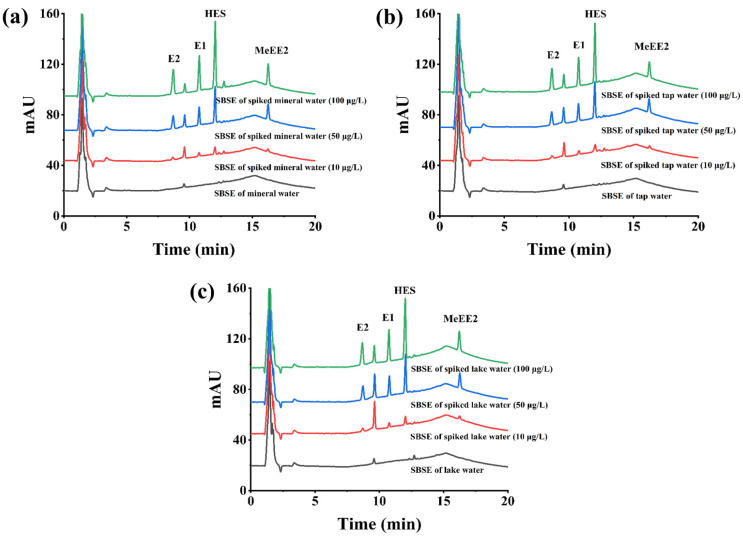
Original and spiked chromatograms of estrogens in mineral water (**a**), tap water (**b**), and lake water (**c**).

The developed TAPB-DHT-COF-coated SBSE-HPLC-DAD method was applied to analyze the presence of four target estrogens in mineral water, tap water, and lake water. The analytical results of the water samples, along with the recoveries of the four estrogens added at different concentration levels, are presented in Table S2. None of the four target estrogens were detected in any of the water samples. The recoveries of the four target estrogens in mineral water, tap water, and lake water ranged from 89.60% to 106.9%, 82.08% to 109.7%, and 92.62% to 109.9%, respectively. Figure 5 illustrates the chromatograms obtained from the analysis of the three environmental water samples. These results indicate that the proposed TAPB-DHT-COF-coated SBSE-HPLC-DAD method has great potential for the accurate analysis of estrogens in environmental water samples.

## 3. Materials and Methods

### 3.1. Synthesis and Preparation of Estrogen Samples

TAPB (purchased from Shanghai Titan Scientific Co., Ltd., Shanghai, China) and DHT (purchased from Shanghai Titan Scientific Co., Ltd., Shanghai, China) were used as monomers in the synthesis process. Additionally, four estrogen standards, E2 (98%), E1 (98%), HES (98%), and MeEE2 (98%), were obtained from Aladdin Company (Shanghai, China). Table S1 provides the structures and other relevant information of these four estrogens.

To prepare the samples for analysis, the four estrogen standards were individually dissolved in acetonitrile to create 1000 mg/L single-component stock solutions, as well as a 1 mg/L mixed-component stock solution. These stock solutions, including both the single-component and mixed-component ones, were stored at 4 °C until further use.

### 3.2. HPLC Analysis of Estrogens

For the determination of estrogens, an Agilent 1260 high-performance liquid chromatography (HPLC) system equipped with a diode array detector (DAD, Agilent, Santa Clara, CA, USA) was utilized. The specific HPLC conditions, reagents, and instruments used in this study are described in detail in the Supplementary Materials (SI).

### 3.3. Synthetic Technique for TAPB-DHT-COF and Product Purification

In a centrifuge tube, TAPB (0.08 mmol, 28.1 mg) and DHT (0.12 mmol, 40.1 mg) were first added. Then, acetonitrile (10.0 mL) was introduced into the tube, and the mixture was sonicated until the monomer was evenly dispersed in the solvent. Afterward, a gradual addition of 0.4 mL of acetic acid (12 M) was performed while continuing the sonication process. This step ensured thorough mixing of the reactants, which were then left to react for 72 h at room temperature. 

Upon completion of the reaction, the mixture was centrifuged at 10,000 rpm for 10 min. The resulting yellow products were subsequently washed three times with tetrahydrofuran and EtOH, followed by drying at 60 °C for 12 h.

### 3.4. Preparation of COF-Coated Stir Bars

In this work, a physical adhesion method was used to prepare the TAPB-DHT-COF-coated stir bars. Detailed procedures can be found in the Supplementary Information (SI).

### 3.5. SBSE Procedure

The sample solutions were prepared in a 20 mL brown vial, which already contained 10 mL of the sample. A COF-coated stir bar was carefully inserted into the vial. For experimental optimization, a concentration of 100 µg/L of the standard solution of estrogens was used. For analytical performance assessments, a range of concentrations from 0.2 to 100 µg/L was employed.

The vial was placed on a stirrer and agitated at a rotational speed of 700 revolutions per minute for 30 min to aid in the extraction process. Afterward, the COF-coated stir bar was gently removed and dried using filter paper.

To facilitate desorption, a custom-made desorption tube was utilized. The tube consisted of a micropipette tip with a capacity of 1000 μL, with the needle tip sealed securely using a flame. Methanol was added to the tube (450 µL) to aid in desorption, and ultrasonication was conducted for 15 min.

Following the desorption process, the stir bars were carefully extracted and cleaned thoroughly with 2 mL of methanol. Ultrasonication was conducted for 5 min to ensure the stir bars were suitable for further use. For future analysis using HPLC, a volume of 20 μL of the supernatant from the desorption solution was extracted.

### 3.6. Water Sample Collection and Processing for Analysis

Mineral water samples were obtained from local supermarkets, tap water was collected from the laboratory, and lake water was gathered from Hubei Minzu University (Enshi, China). For the analysis, the mineral water samples and tap water samples were directly subjected to solid-phase microextraction (SBSE). On the other hand, the lake water underwent a preliminary filtration step using a 0.45-µm polytetrafluoroethylene (PTFE) filter membrane. To ensure the stability of the samples, they were stored at 4 °C until further use. Subsequently, 10 mL of each prepared water solution was extracted and desorbed according to the procedure described in Section 2.5. The extracted samples were then subjected to high-performance liquid chromatography with diode array detection (HPLC-DAD).

## 4. Conclusions

The aim of this study was to synthesize a covalent organic framework (COF) named TAPB-DHT-COF using 1,3,5-tris(4-aminophenyl)benzene and 2,5-bis(hexyloxy)terephthalaldehyde as precursor materials. In addition, a TAPB-DHT-COF-coated stir bar was produced using an adhesion approach. This innovative method involved the use of a TAPB-DHT-COF-coated stir bar solid-phase microextraction (SBSE) technique coupled with high-performance liquid chromatography with diode array detection (HPLC-DAD) to analyze four endocrine disruptor compounds (estrogens) in various water samples, including bottled mineral water, tap water, and lake water.

This approach provided several advantages, including exceptional selectivity, high sensitivity, and a low limit of detection. It is hypothesized that the COF coating on the stir bar facilitated the adsorption of the target estrogens, possibly through hydrogen bonding and π–π interactions. The use of a self-produced stir bar coated with COF compounds has expanded the potential applications of COF compounds in SBSE techniques. This development has demonstrated the significant potential for studying estrogens in water samples under ambient conditions.

Overall, the successful synthesis of TAPB-DHT-COF and the development of a TAPB-DHT-COF-coated SBSE-HPLC-DAD method offer great potential for the analysis of estrogens in various water sources. The unique properties of COF compounds make them a promising material for environmental monitoring and analysis.

## Figures and Tables

**Table 1:** Preparation reproducibility of the TAPB-DHT-COF stir bar.

Estrogen	RSDs (%)	
	Bar to Bar (*n* = 6)	Batch to Batch (*n* = 5)
E2	7.43	10.8
E1	4.65	9.37
HES	3.71	4.25
MeEE2	4.35	6.11

**Table 2:** Analytical performance of the TAPB-DHT-COF-coated SBSE-HPLC-DAD method.

Compounds	Linear Range (µg/L)	Linear Equation	R2	Limits of Detection (µg/L)	RSD% *n* = 8	Enrichment Factor
E2	0.5–100	y = 1.982x + 1.336	0.9993	0.15	4.25	49
E1	0.5–100	y = 2.041x + 3.232	0.9991	0.15	3.78	40
HES	0.2–100	y = 1.997x + 3.759	0.9996	0.06	3.40	47
MeEE2	0.5–100	y = 2.423x − 7.531	0.9997	0.15	1.80	39

**Table 3:** Comparison with current analytical methods for the determination of estrogen in environmental samples.

Methods	Extraction Time (min)	LODs (µg/L)	RSD%	Samples	Ref.
VA-ME-HPLC-FLD	30	0.005–1	≤11.4	urine	[37]
IOT-SPME-HPLC-DAD	20	0.21–0.80	2.4–6.6	environmental water	[38]
DLLME-HPLC-UV	-	30–70	0.6–4.2	environmental aquatic	[39]
SPE-HPLC-UV	60	0.18	<4.4	milk power, drinking water	[40]
SPME-HPLC-UV	20	1.5–5.5	4.0–6.1	milk power	[41]
SBSE-HPLC-UV	55	0.3–0.4	≤16	water	[42]
SBSE-HPLC-UV	20	0.21–1.6	8.4–11	pork, chicken	[43]
SBSE-HPLC-UV	120	0.3–1.0	2.1–17.1	water, urine	[44]
SBSE-HPLC-UV	40	0.11–0.31	-	water, chicken, pork	[45]
SBSE-HPLC-DAD	30	0.06–0.15	1.8–4.25	water	this work

FLD: Fluorescence Detector. UV: Ultraviolet detector.

## Data Availability

Data are available on request from the corresponding author.

## Supplementary Materials

The following supporting information can be downloaded at: https://www.mdpi.com/article/10.3390/molecules29235763/s1, Reagents and instruments used in the experiment; Preparation of the stir bar; Enrichment factor calculation process; Table S1: Structures, logKo/w, and pKa values of four estrogen compounds; Table S2: Recoveries for the three environmental water samples (mean ± SD, *n* = 3); Figure S1: Effects of desorption solvent on the desorption of estrogens; Figure S2: Effects of desorption volume on the desorption of estrogens; Figure S3: Effects of stirring rate on the extraction of estrogens; Figure S4: Effects of extraction time on the extraction of estrogens; Figure S5: Effects of desorption time on the desorption of estrogens; Figure S6: Effects of salt concentration on the extraction of estrogens; Figure S7: Effects of sample solution pH on the extraction of estrogens; Figure S8: Lifetime of TAPB-DHT-COF-coated stir bars; Figure S9: Effect of PDMS glue on estrogen adsorption; Figure S10: The optimal configurations of the stable complexes.
