# Supplementary material for: Development and Application of a Robust Imine-Based Covalent Organic Framework for Stir Bar Sorptive Extraction of Estrogens in Environmental Water
Source: Molecules. 2024 Dec 6;29(23):5763. doi: 10.3390/molecules29235763 (PMC11643056; doi:10.3390/molecules29235763)
Supplement: Supplementary file 1 [file molecules-29-05763-s001.zip › molecules-3313993-supplementary.pdf]

Development and Application of a Robust Imine-Based Covalent Organic Framework for Stir Bar Sorptive Extraction of Estrogens in Environmental Water

Jianing Sun, Xixi Lian, Lianzhi Wang * and Zhengchao Duan *

School of Chemistry and Environmental Engineering, Hubei Minzu University, Enshi 445000, China; sjn_ing@163.com (J.S.); liana190203@163.com (X.L.)
* Correspondence: 1998032@hbmzu.edu.cn (L.W.); 1997015@hbmzu.edu.cn (Z.D.)

Supplementary materials

Reagents and instruments used in the experiment.

Preparation of the stir bar.

Enrichment factor calculation process.

Theoretical calculation.

Table S1 Structures, log$K_{o/w}$, and pK_a values of four estrogen compounds.

Table S2 Recoveries for the three environmental water samples (mean±SD, n = 3).

Fig. S1 Effects of desorption solvent on the desorption of estrogens.

Fig. S2 Effects of desorption volume on the desorption of estrogens.

Fig. S3 Effects of stirring rate on the extraction of estrogens.

Fig. S4 Effects of extraction time on the extraction of estrogens.

Fig. S5 Effects of desorption time on the desorption of estrogens.

Fig. S6 Effects of salt concentration on the extraction of estrogens.

Fig. S7 Effects of sample solution pH on the extraction of estrogens.

Fig. S8 Lifetime of TAPB-DHT-COF-coated stir bars.

Fig. S9 Effect of PDMS glue on estrogen adsorption.

Fig. S10 The optimal configurations of the stable complexes.

Reagents, instruments and HPLC condition used in the experiment.

Sodium hydroxide, sodium chloride, hydrochloric acid, acetic acid, methanol, ethanol, and acetone were purchased from Sinopharm Chemical Reagent Co., Ltd (Shanghai, China). Tetrahydrofuran (THF) was purchased from Shanghai Macklin Biochemical Co., Ltd (Shanghai, China). Deionized water was used throughout the experiments from a water purifying instrument from Chengdu Haokang Technology Co., Ltd (Chengdu, China). The above chemicals and reagents were of analytical grade, and the acetonitrile used for the HPLC assays was of HPLC grade and purchased from Thermo Fisher Scientific (China) (Shanghai, China). Wahaha purified water was purchased from Wahaha Group Limited (Hangzhou, China).

A Nicolet Avatar Model 370 Fourier-transform infrared (FT-IR) spectrometer (Thermo, Waltham, USA) was used for the structural characterization of the COF materials. An X-ray diffraction (XRD)-7000 X-ray diffractometer (Shimadzu, Kyoto, Japan) was used for the physical phase and crystal structure analysis of the COF materials. A TG/DTA 7300 synchronous thermal analyzer (Hitachi High-Tech, Tokyo, Japan) was used to analyze the thermal properties of the COF materials. Scanning electron microscopy (SEM) was performed with a Gemini 300G scanning electron microscope (Carl Zeiss AG, Oberkochen, Germany) and an accelerating voltage of 5 kV was used to characterize the surface morphology and coating thickness of the stir bar coating. A JEOL-794 transmission electron microscope (JEOL Corporation, Tokyo, Japan) was used to observe the fine structure of the stir bar coating. An Autosorb-iQ fully automatic specific surface area and pore size distribution analyzer (Quantachrome, Florida, USA) was used to analyze specific surface area and pore size distribution of the COF materials at 77 K after being degassed at 120°C for 8 h. An IKA color squid white magnetic stirring apparatus (Baden-Württemberg, Germany) was adopted for the extraction processes. A KQ-400B ultrasonic cleaner (Kunshan Ultrasonic Instruments Co., Ltd., Kunshan, China) was used for ultrasonic desorption and activation of the stir bars. A magnetic PHS-25 digital pH meter (Shanghai Yidian

Scientific Instruments Co., Ltd., Shanghai, China) was used to adjust the pH value of samples.

An Agilent 1260 HPLC (Agilent, USA) with a DAD-detector and a C18 column (Agilent, Santa Clara, CA, USA; 4.6 mm×150 mm, 5 μm) was adopted for the determination of estrogens. The mobile phase was eluted in a gradient using the following procedure: 0–5 min, acetonitrile/water (38/62, v/v); 5.01–13 min, acetonitrile/water (70/30, v/v); and 13.01–20 min, acetonitrile/water (38/62, v/v). The injection volume was 20 μL, the flow rate was 1 mL/min, and the detection wavelength was 200 nm.

Preparation of the stir bar.

One iron wire was cut into 1.5 cm and put in a 2 cm capillary glass tube. It was made into a "dumbbell-shaped" glass stir bar by burning small bubbles at the ends of a capillary glass tube with an alcohol lamp. Then, the glass stir bar was activated in 1 mol/L NaOH for 24 h, and then washed with 1 mol/L HCl and deionized water until neutral[34].

In this work, a physical adhesion method was used to prepare the TAPB-DHT-COF-coated stir bars. First, the synthesized TAPB-DHT-COF was ground evenly and placed in a clean petri dish. Then, the dumbbell-shaped glass stir bar was submerged in PDMS glue (Glue A/Glue B, 10/1) to evenly coat with a layer of adhesive. After that, the glass stir bar with PDMS glue was removed and placed in a petri dish containing TAPB-DHT-COF powder and rolled back and forth until the TAPB-DHT-COF powder adhered evenly to the entire glass stir bar. Then, the TAPB-DHT-COF-coated stir bar was removed and placed in an oven at 60°C for aging for 12 h. The stir bar was ultrasonically washed with methanol until the solvent became colorless to remove impurities in the COF coating material before use.

Enrichment Factor calculation process.

The calibration curves with SBSE process are called the working curves in the following context. Specifically, a series of standard solutions of four estrogens with

different concentrations (0.1-200 μg/L) were prepared and subjected to the proposed SBSE process and HPLC-DAD detection; the working curves were obtained by plotting the signal intensity (peak areas in HPLC-DAD as Y-axis) of each estrogens to their corresponding concentrations (X-axis). The slope of the working curves (corresponding to the sensitivity of the developed SBSE-HPLC-DAD method) was then obtained in the linear equation. The calibration curves without SBSE process were obtained by preparing a series of standard solution of four estrogens with different concentrations (0.01-20 mg/L) and directly subjecting them to HPLC-DAD analysis; the calibration curves were obtained by plotting the signal intensity (peak areas in HPLC-DAD as Y-axis) of each estrogens to their corresponding concentrations (X-axis), and the slopes correspond to the sensitivity of the HPLC-DAD method. Then, EF was calculated based on the slope ratio of the working curves with SBSE and calibration curves without the SBSE process.

Theoretical calculation

Theoretical calculation was carried out on the Materials Studio 7.0 software. The structures of COF and four estrogens were adjusted through the Dmol3 module. Afterward, a 2×2×6 COF unit cell was established to simulate the adsorption conformation of four target molecules. In order to save computing resources, we used pure estrogen molecules instead of solutions for simulation. The adsorption energies of the molecule in F-COF (E_{ads}) were calculated by the following formula:

$E_{ads} = E_{COF-estrogen} - (E_{COF} + E_{estrogen})$, of which E_{COF} and $E_{estrogen}$ referred to the energies of optimal COF and estrogen, respectively. $E_{COF-estrogen}$ was the total energy of the optimal absorption model. It could be assumed that the adsorption process was spontaneous and exothermic when the value of Eads was negative. By the way, the more negative the Eads value, the stronger the adsorption affinity.

Table S1 Structures, log*P*, and p*K*a values of four estrogen compounds.

Compounds	Structure	pKa	Log P
**E2**	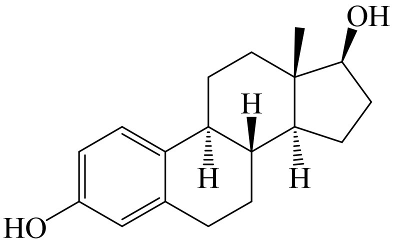	10.46	4.13
**E1**	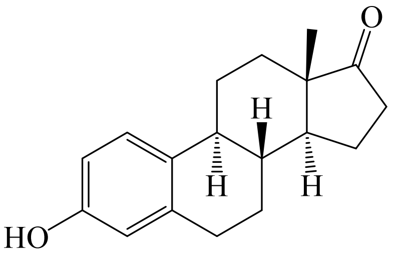	10.34	3.68
**HES**	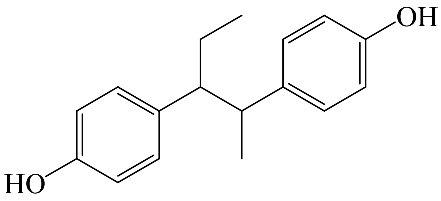	9.80	4.98
**MeEE2**	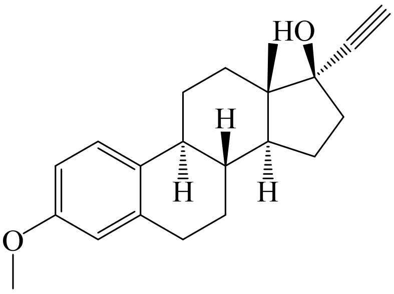	13.10	5.17

Table S2

Recoveries for the three environmental water samples (mean±SD, n = 3)

Analytes	Mineral water			Tap water			Lake water		
	Added (μg/L)	Found (μg/L)	Recovery(%)	Added (μg/L)	Found (μg/L)	Recovery(%)	Added (μg/L)	Found (μg/L)	Recovery(%)
E2	0	ND	ND	0	ND	ND	0	ND	ND
	10	8.960±0.34	89.60	10	10.07±0.63	100.7	10	10.99±0.57	109.9
	50	51.56±1.32	103.1	50	46.90±4.33	93.80	50	52.31±1.35	104.6
	100	101.0±2.9	101.0	100	89.06±2.93	89.06	100	93.73±2.53	93.73
E1	0	ND	ND	0	ND	ND	0	ND	ND
	10	9.390±0.68	93.90	10	10.05±0.47	100.5	10	10.17±0.72	101.7
	50	53.43±1.6	106.9	50	46.11±4.49	92.22	50	54.75±1.23	109.5
	100	106.9±3.25	106.9	100	89.49±4.69	89.49	100	99.7±2.44	99.70
HES	0	ND	ND	0	ND	ND	0	ND	ND
	10	10.54±0.14	105.4	10	10.63±0.09	106.30	10	10.52±0.4	105.2
	50	53.92±0.53	107.8	50	47.94±4.55	95.88	50	54.55±1.1	109.1
	100	107.1±3.18	107.1	100	92.95±4.18	92.95	100	94.75±2.92	94.75
MeEE2	0	ND	ND	0	ND	ND	0	ND	ND
	10	10.20±0.24	102.0	10	10.97±0.26	109.70	10	10.61±0.41	106.1
	50	49.49±2.18	98.98	50	41.04±2.34	82.08	50	46.31±1.87	92.62
	100	99.42±1.4	99.42	100	96.67±2.55	96.67	100	94.88±2.94	94.88

s.d.: standard deviation

ND: not detected

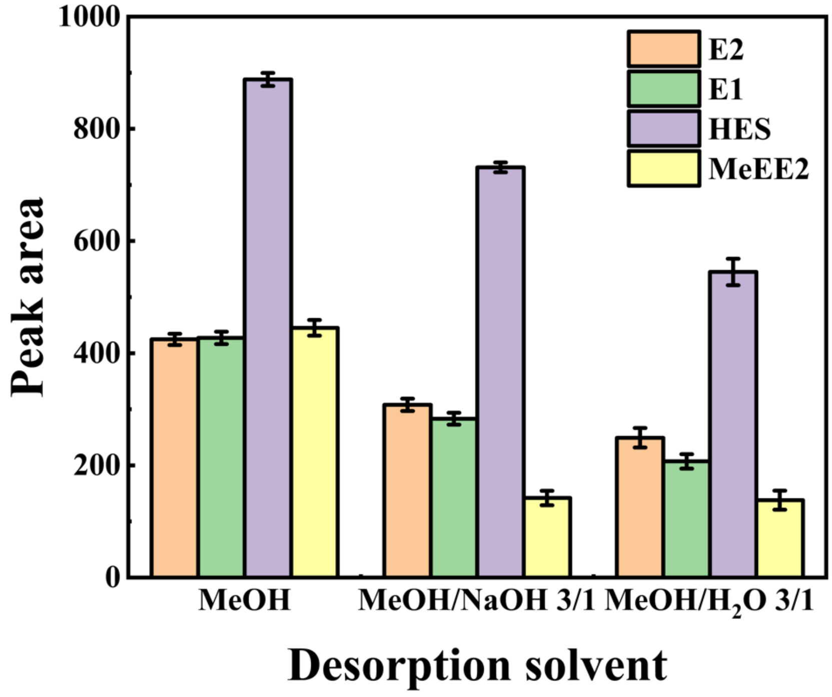

Fig. S1 Effects of desorption solvent on the desorption of estrogens. Conditions: no salt addition; no sample pH adjustment required; stirring rate and extraction time, 600 rpm and 30 min; desorption time, 10 min; desorption volume of 200 μL.

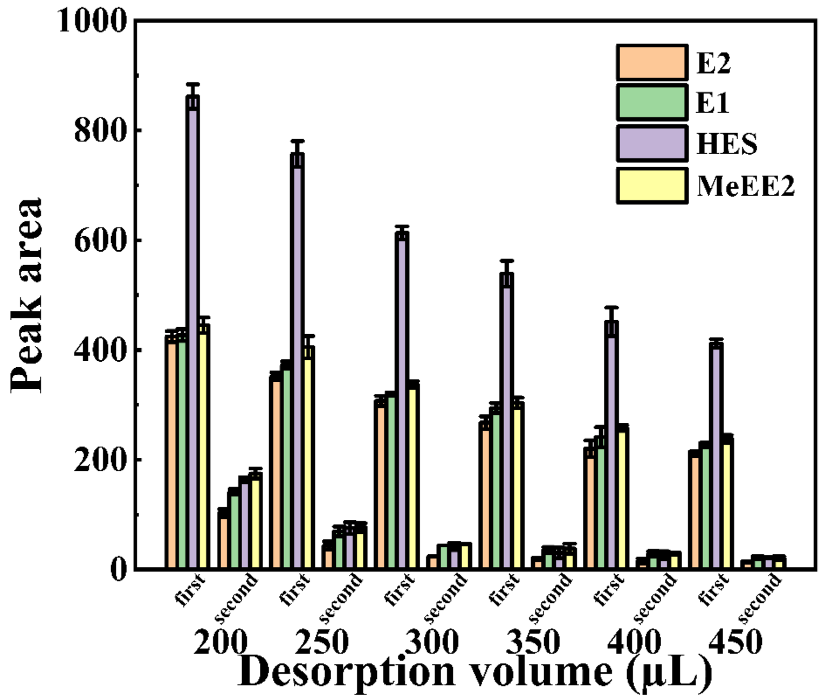

Fig. S2 Effects of desorption volume on the desorption of estrogens. Conditions: no salt addition; no sample pH adjustment required; stirring rate and extraction time, 600 rpm and 30 min; desorption solvent and time, MeOH and 10 min.

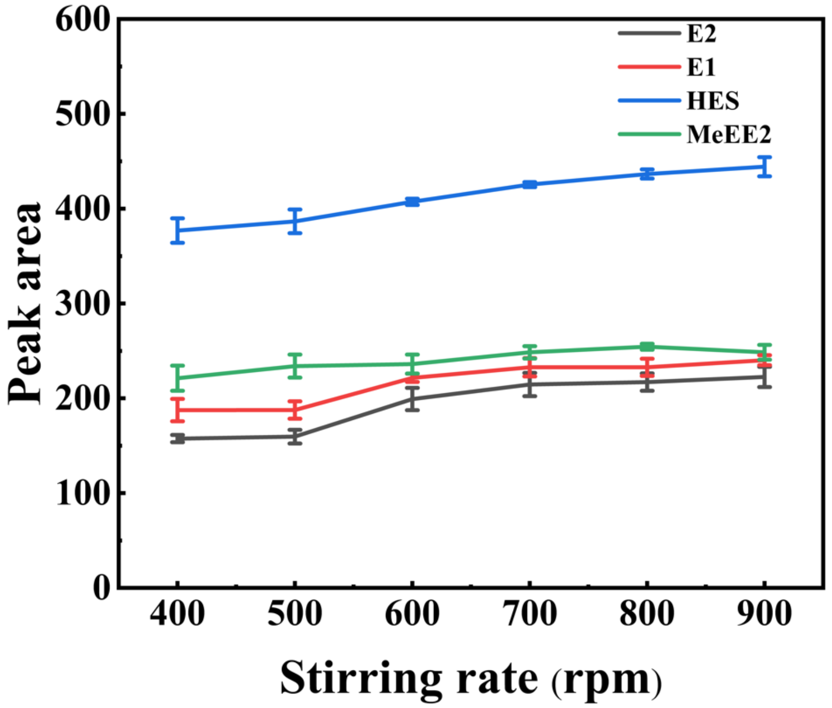

Fig. S3 Effects of stirring rate on the extraction of estrogens. Conditions: no salt addition; no sample pH adjustment required; extraction time, 30 min; desorption solvent and time, MeOH and 10 min; desorption volume of 450 μL.

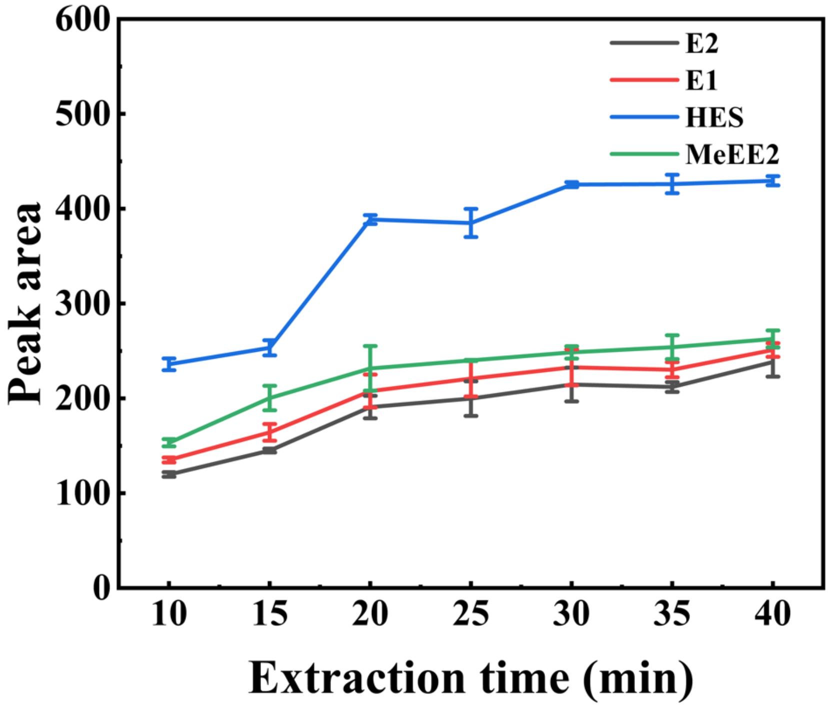

Fig. S4 Effects of extraction time on the extraction of estrogens. Conditions: no salt addition; no sample pH adjustment required; stirring rate, 700 rpm; desorption solvent and time, MeOH and 10 min; desorption volume of 450 μL.

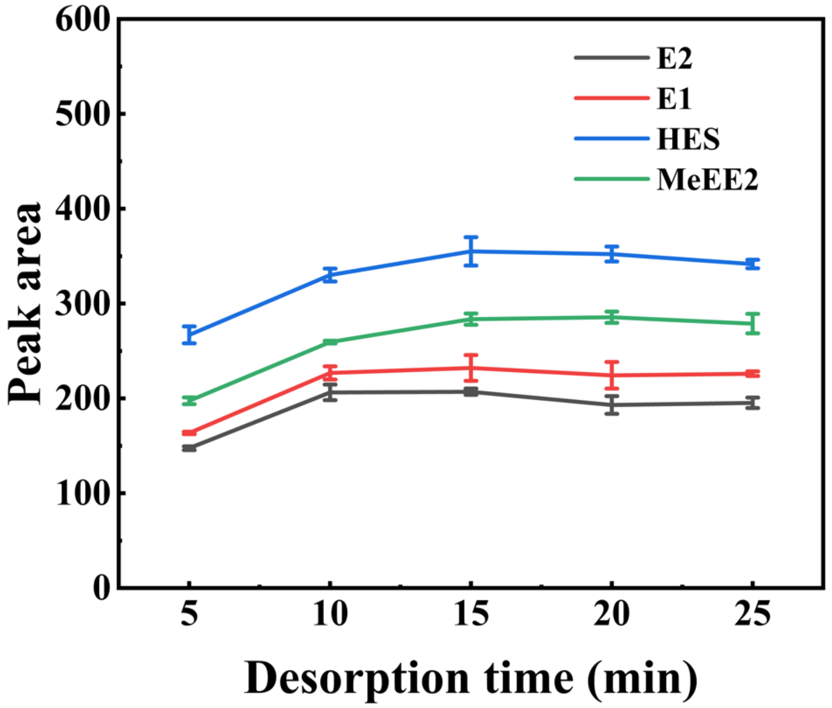

Fig. S5 Effects of desorption time on the desorption of estrogens. Conditions: no salt addition; no sample pH adjustment required; stirring rate and extraction time, 700 rpm and 30 min; desorption solvent, MeOH; desorption volume of 450 μL.

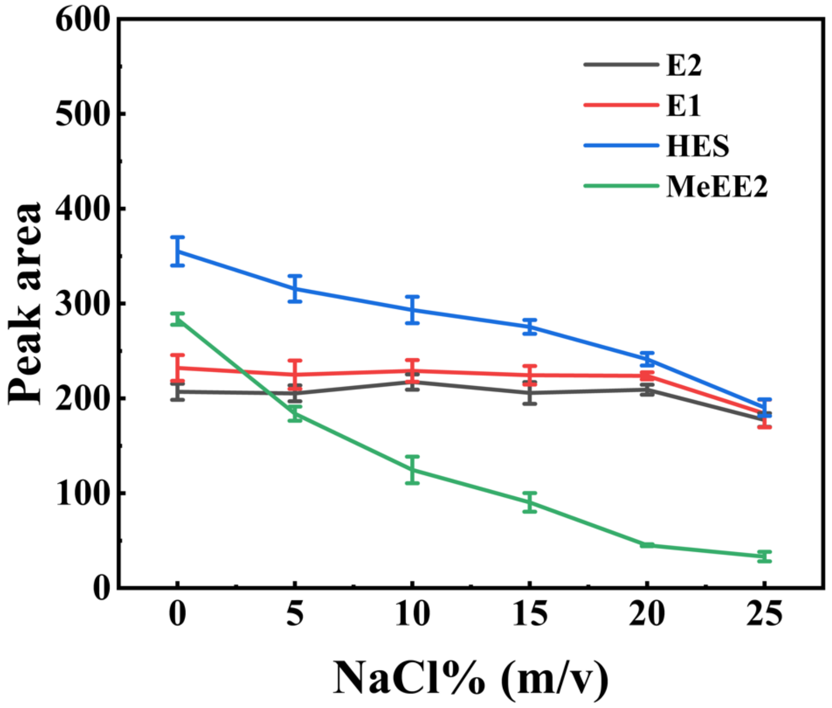

Fig. S6 Effects of salt concentration on the extraction of estrogens. Conditions: No sample pH adjustment required; stirring rate and extraction time, 700 rpm and 30 min; desorption solvent and time, MeOH and 15 min; desorption volume of 450 μL.

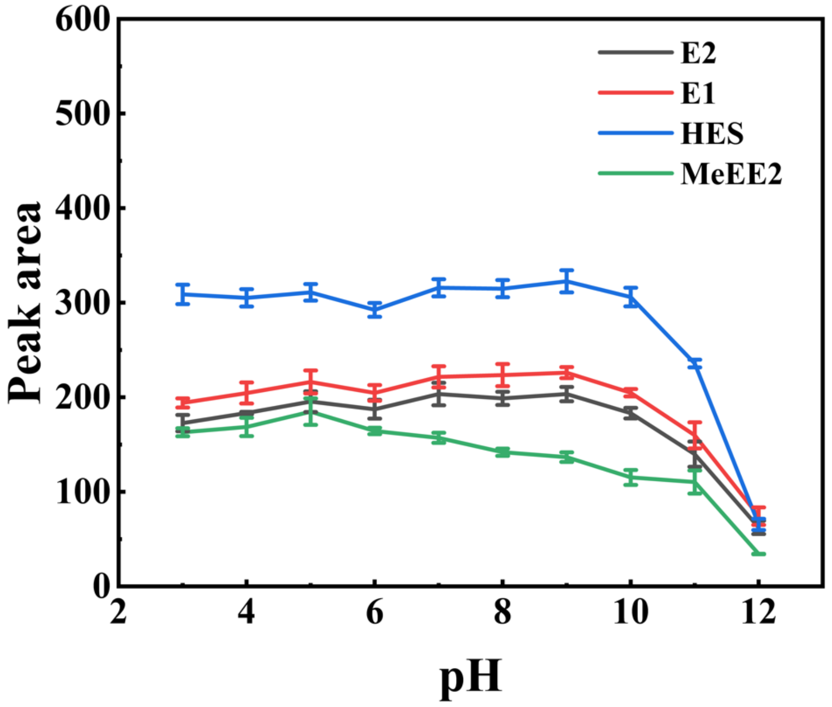

Fig. S7 Effects of sample solution pH on the extraction of estrogens. Conditions: no salt addition; stirring rate and extraction time, 700 rpm and 30 min; desorption solvent and time, MeOH and 15 min; desorption volume of 450 μL.

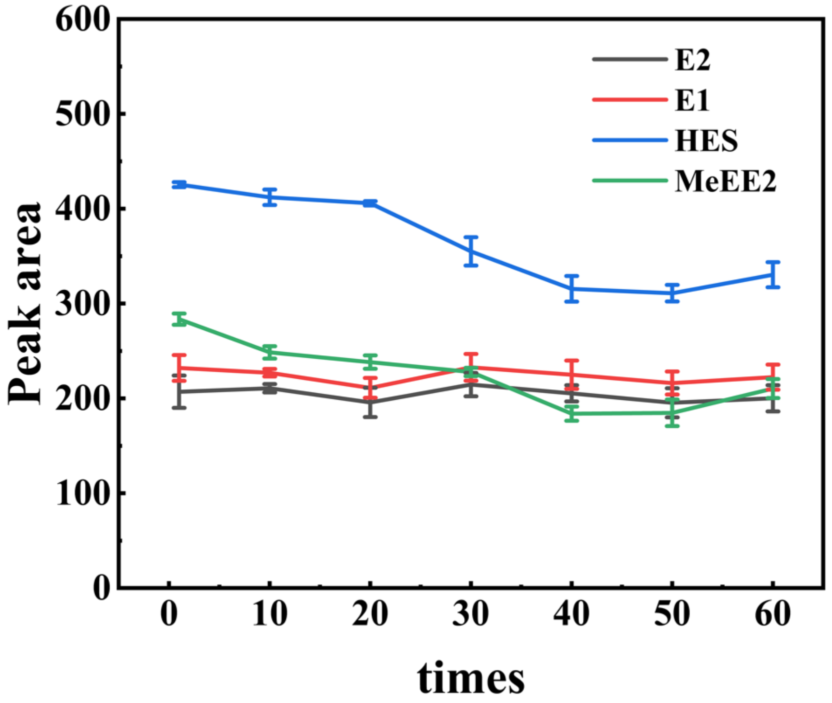

Fig. S8 Lifetime of the TAPB-DHT-COF-coated stir bars. Conditions: no salt addition; no sample pH adjustment required; stirring rate and extraction time, 700 rpm and 30 min; desorption solvent and time, MeOH and 15 min; desorption volume of 450 μL.

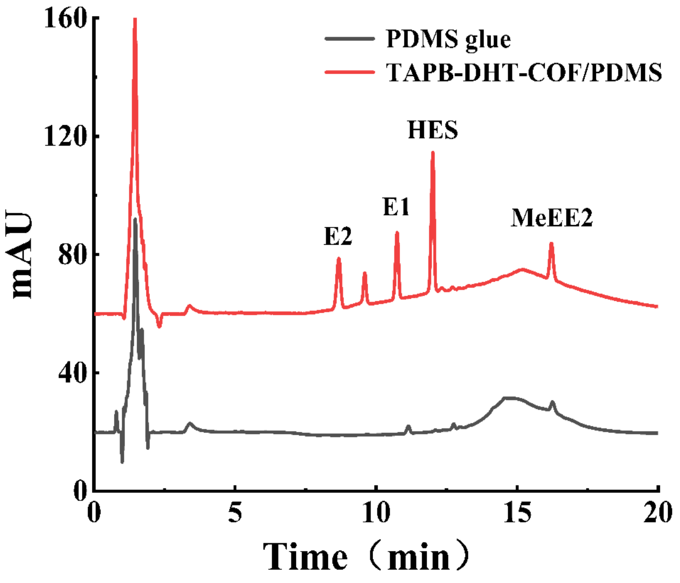

Fig. S9 Comparison chromatogram of PDMS glue and TAPB-DHT-COF adsorption

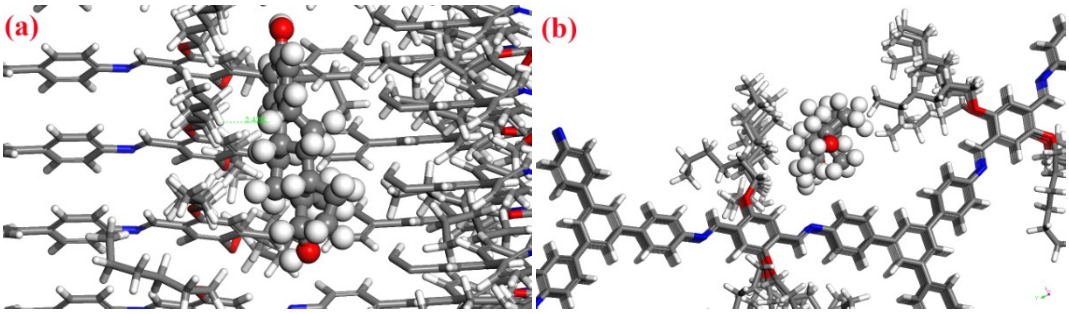

Fig. S10 The optimal configurations of the stable complexes (side view (a) and over view (b)).
